# Silver nanoparticles promote the emergence of heterogeneic human neutrophil sub-populations

**DOI:** 10.1038/s41598-018-25854-2

**Published:** 2018-05-14

**Authors:** Jennifer A. Fraser, Sadie Kemp, Lesley Young, Mark Ross, Morag Prach, Gary R. Hutchison, Eva Malone

**Affiliations:** School of Applied Sciences, Edinburgh Napier University, Sighthill Campus, Edinburgh, EH11 4BN UK

## Abstract

Neutrophil surveillance is central to nanoparticle clearance. Silver nanoparticles (AgNP) have numerous uses, however conflicting evidence exists as to their impact on neutrophils and whether they trigger damaging inflammation. Neutrophil’s importance in innate defence and regulating immune networks mean it’s essential we understand AgNP’s impact on neutrophil function. Human neutrophil viability following AgNP or Ag Bulk treatment was analysed by flow cytometry and AnV/PI staining. Whilst AgNP exposure did not increase the total number of apoptotic neutrophils, the number of late apoptotic neutrophils was increased, suggesting AgNP increase transit through apoptosis. Mature (CD16^bright^/CD62L^bright^), immature (CD16^dim^/CD62L^bright^) and apoptotic (CD16^dim^/CD62L^dim^) neutrophil populations were evident within isolated neutrophil preparations. AgNP exposure significantly reduced CD62L staining of CD16^bright^/CD62L^bright^ neutrophils, and increased CD16 staining of CD16^dim^/CD62L^bright^ populations, suggesting AgNPs trigger neutrophil activation and maturation, respectively. AgNP exposure dramatically increased IL-8, yet not classical pro-inflammatory cytokine release, suggesting AgNP triggers neutrophil activation, without pro-inflammation or damaging, necrotic cell death. For the first time, we show AgNPs differentially affect distinct sub-populations of circulating human neutrophils; activating mature neutrophils with the emergence of CD16^bright^/CD62L^dim^ neutrophils. This may stimulate particle clearance without harmful inflammation, challenging previous assumptions that silver nanomaterials induce neutrophil toxicity and damaging inflammatory responses.

## Introduction

Analyses of the impact of particles, either nanoparticle or air pollution, on biological systems have recognised the importance of neutrophil surveillance in particle clearance. Indeed, neutrophil recruitment has been used as a measure of inflammation following nanomaterial exposure in rodents^1,2^.

As the first line of defence against invading pathogens, circulating neutrophils are rapidly recruited to sites of inflammation associated with infection or particle exposure^2^. Here they become activated, and phagocytose and destroy microbes by releasing proteases, cationic defence peptides and reactive oxygen species^3^. Activated neutrophils release an array of cytokines and chemokines^4^ which play a fundamental role in modulating immune cell networks and orchestrating the adaptive immune response^5^. Due to their potency once activated, inappropriate neutrophil recruitment or activation can contribute to inflammatory pathogenesis and disease^6^.

The view of neutrophils as a terminally differentiated, homogenous effector cells is rapidly changing as evidence of neutrophil heterogeneity and phenotypic diversity has emerged^7^. Billions of neutrophils are mobilised from the bone marrow daily under normal homeostasis, and peripheral blood contains a heterogeneous population of neutrophils at various stages of maturity, mobility and activity^8^. These can be distinguished by their differential expression of cell surface markers. Immature neutrophils express low levels of the FcγRIII receptor (CD16) and high levels of L-selectin (CD62L)^8^. CD16 expression increases as neutrophils differentiate, therefore CD16 status indicates neutrophil maturation^9^. CD62L expression is gradually lost as neutrophils transit the circulation and age, and CD62L^dim^ neutrophils are preferentially eliminated from the circulation by macrophage clearance in the spleen, liver and bone marrow^8,10^. This contributes to a neutrophil’s short half-life (~14 hours – 3 days)^11,12^ and limits their potential to cause unwanted toxicity^12^. CD62L expression is also lost in response to microbial peptides^13^, therefore CD62L status is indicative of neutrophil activation and age. The integrin adhesion molecule, CD11b, is dramatically upregulated upon neutrophil activation, therefore aged and activated neutrophil populations can be distinguished, *in vitro*, via their CD16, CD62L and CD11b status and flow cytometry^13,14^.

Distinct sub-populations of circulating neutrophils have been found in acute systemic inflammation, and differences in phenotypic function within these sub-populations has been identified^15^. CD16^bright^/CD62L^dim^ neutrophils are associated with a lower phagocytic index and reactive oxygen species production compared to normal, mature CD16^bright^/CD62L^bright^ neutrophils^16^ and have decreased adhesion to endothelium under flow conditions, suggesting attenuated extravasation capacity^14^. CD16^bright^/CD62L^dim^ neutrophils can also suppress T-cell proliferation *in vitro*^15,17^ suggesting CD16^bright^/CD62L^dim^ neutrophils display immunosuppressive properties. Plasticity in neutrophil function and phenotype has been documented, and the prevalence of neutrophils in different sub-populations can alter in response to environmental factors (reviewed by^7^); neutrophils are clearly more versatile and dynamic than previously anticipated.

Worldwide silver nanoparticle use is rising faster than any other nanomaterial^18^, making our exposure to them in daily life, through consumption or environmental contact, ever more likely. Potent antimicrobial properties^19^ have made silver nanoparticle an attractive component in medical devices and wound dressings^18^. Rodent models demonstrate evidence of nanoparticle deposits in a variety tissues, including the primary site of exposure, and their translocation and deposition in secondary sites in certain situations^20,21^. Neutrophil infiltration to the lung and peritoneal cavities is shown in rodent models 24 hours after instillation or injection of silver particles^20,22^. In other nanoparticle studies, this is correlated with toxicity and inflammation^23^. There is however conflicting evidence on the impact of silver on neutrophil function, and whether silver nanoparticles are toxic to neutrophils and trigger damaging inflammation. Due to their importance in innate host defence, and as a key regulator of immune networks and inflammatory pathogenesis^5^, it is essential we understand the impact of silver nanoparticles on neutrophil function.

Isolated neutrophils and neutrophil-like models, derived from differentiation of immortalised cell lines, have been used to draw parallels with the observations made, *in vivo* and literature to date has focussed on key endpoints of neutrophil death and inflammatory cytokine release. However, in light of advances in understanding neutrophil heterogeneity and functional plasticity, this approach may limit our understanding of what is actually occurring and underestimate the complexity of the neutrophil response, *in vivo*.

In this study, we further elucidate human neutrophil’s response to silver nanoparticle exposure, beyond the traditional global assessment of inflammation and cell death. We investigated the impact of silver nanoparticles on circulating human neutrophil heterogeneity and the emergence of neutrophil subsets, by assessing CD16 and CD62L cell surface expression. Our data shows silver particles promote the activation of mature circulating neutrophils and maturation of immature neutrophils and is associated with apoptotic, rather than damaging pro-inflammatory, necrotic cell death, challenging previous assumptions on silver nanomaterial-induced inflammation and toxicity.

## Results

### Silver nanoparticles alter neutrophil size and granularity

**Figure 1:**
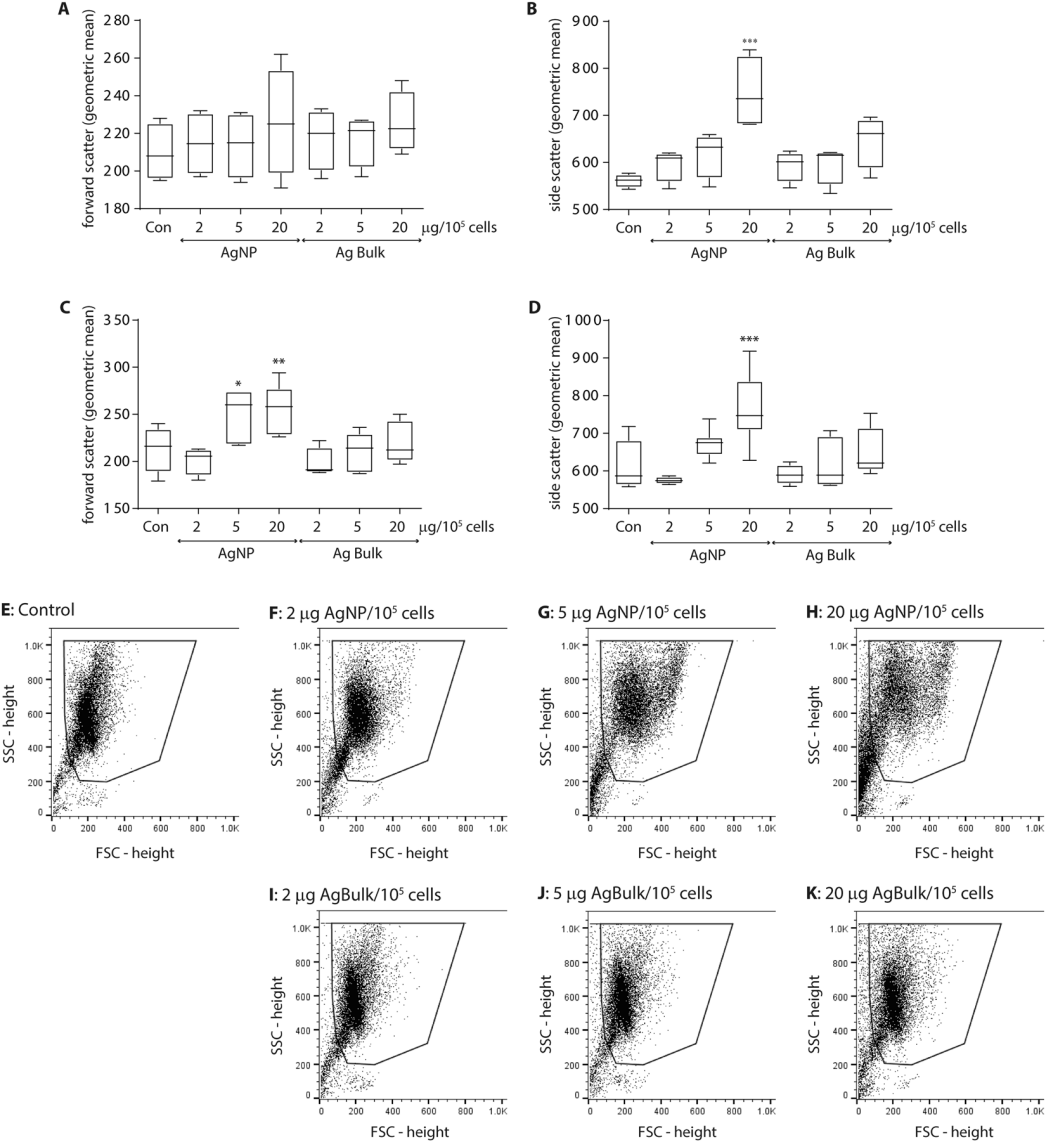
Exposure to silver nanoparticles increases human neutrophil size and granularity. Human neutrophils were exposed to 2, 5 or 20 µg silver nanoparticles (AgNP) or bulk particles (Ag Bulk) per 10^5^ cells at 37 °C and analysed by flow cytometry analysis, counting 10000 gated events. Neutrophil forward (**A** and **C**) and side scattering (**B** and **D**) after 4 hr (**A** and **B**) and 20 hr (**C** and **D**) was quantified. Plots present the maximum and minimum geometric mean for n > 4 preparations; the median size is indicated by the horizontal bar. *p < 0.05; **p < 0.01; ***p < 0.001. Representative flow cytometry profiles of human neutrophil forward (FSC) and side (SSC) scattering at 20 hr in untreated controls (**E**) or neutrophils exposed to 2, 5 or 20 µg AgNP (**F**–**H**) or Ag Bulk particles (**I**–**K**). The gated region is indicated.

Human neutrophils were exposed to low, medium and high concentrations of silver (Ag) nanoparticles (AgNP) for 4 and 20 hours and neutrophil size (forward scatter) and granularity (side scatter) was assessed by flow cytometry. Ag Bulk particles were included as a control. Scatter characteristics of AgNP and Ag Bulk particles alone were determined and these events were excluded from analysis by gating (see Fig. 1)

Little difference in neutrophil size was observed 4 hours after AgNP (209.75 ± 15.48 vs 225.75 ± 29.10) or Ag Bulk (209.75 ± 15.48 vs 225.5 ± 16.42) treatment (Fig. 1A), however AgNP caused a concentration dependant increase in neutrophil granularity (Fig. 1B), increasing side scattering from 561.25 ± 14.24 to 747.75 ± 78.73 (p = 0.0001). High concentrations of Ag Bulk caused a small, yet insignificant increase in neutrophil granularity (561.25 ± 14.24 vs 646.50 ± 56.10) (Fig. 1B, p = 0.0866). Neutrophil size was unchanged after 20 hours in culture (Fig. 1C vs A; 1E; 209.75 ± 15.48 vs 211.29 ± 22.82) however granularity increased from 561.25 ± 14.24 to 610.86 ± 63.62 (Fig. 1D vs B). AgNP caused a further increase in neutrophil size and granularity at 20 hours (Fig. 1C,D,F–H) increasing forward and side scattering to 241.86 ± 40.76 (Fig. 1C, p = 0.003) and 758.71 ± 94.54 (Fig. 1D and H; p = 0.0004) respectively. Increased neutrophil size or granularity was not observed in the presence of Ag Bulk after 20 hours (Fig. 1C and D,I–K). These findings show AgNP trigger significant morphological changes in human neutrophils. The lack of significant effect of Ag Bulk particles indicates these changes are due to the particles’ nanoproperties.

### Prolonged exposure to AgNP attenuates neutrophil viability

**Figure 2:**
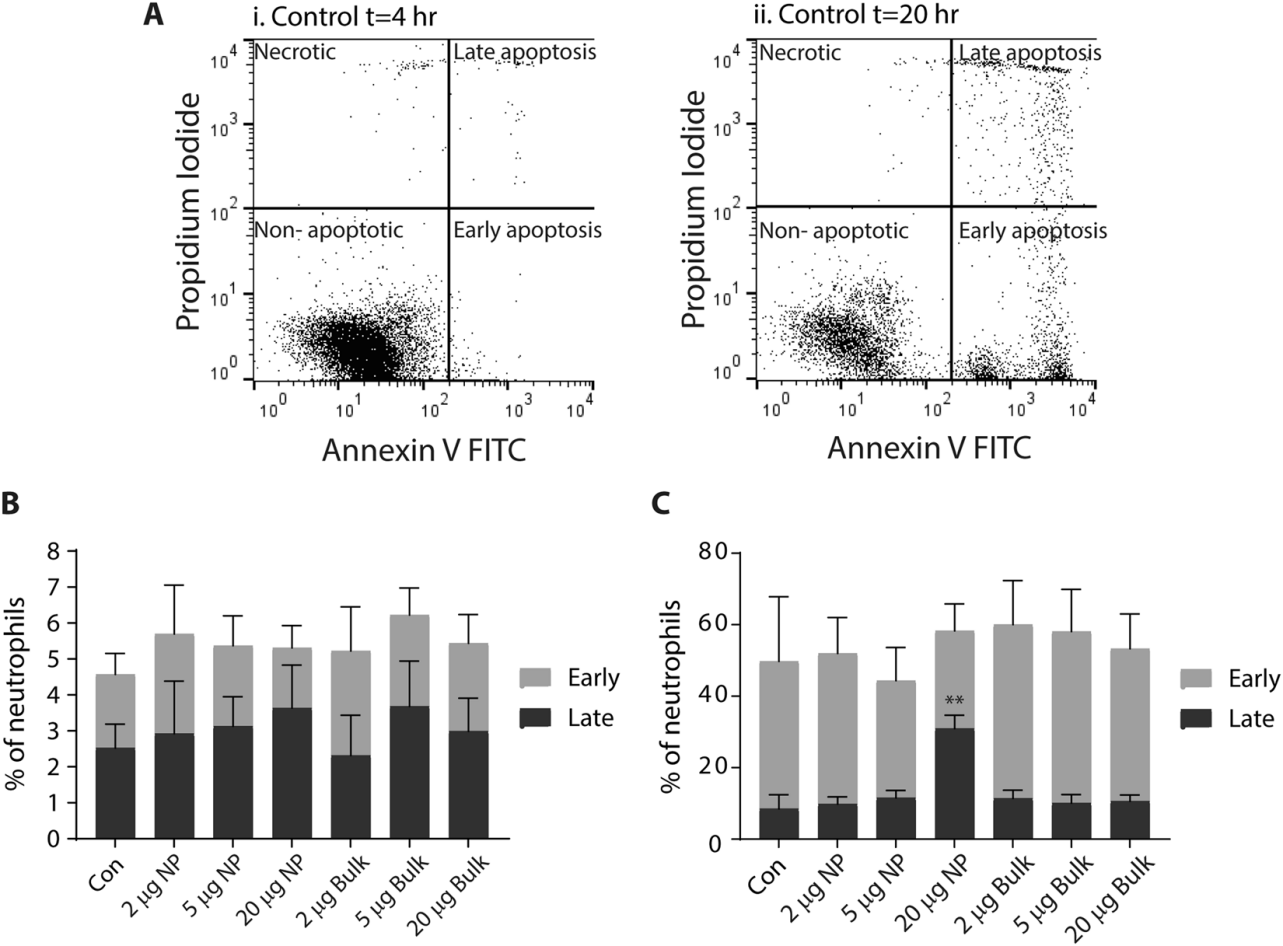
The impact of silver nanoparticles on human neutrophil viability. (**A**) Representative dot plots obtained from AnnexinV-FITC/Propidium iodide staining of neutrophil viability after T = 4 hr (panel i) and 20 hr (panel ii) in culture. The position of the quadrants and the type of cell death depicted in each quadrant is indicated. (**B** and **C**) Human neutrophils were untreated (Con) or exposed to 2, 5 or 20 µg AgNP (NP) or Ag Bulk particles per 10^5^ cells for 4 hr and 20 hr at 37 °C before viability was assessed via AnV and PI staining and flow cytometry, counting 10000 gated events. The number of events per quadrant was calculated as a percentage of total number of gated events and expressed as the percentage of early (AnV^+ve^/PI^−ve^) or late (AnV^+ve^/PI^+ve^) stage apoptosis at either 4 hr (**B**) and 20 hr (**C**); Data is presented as the mean percentage ± SEM where n = 3–4; **p < 0.01.

AgNP’s impact on neutrophil viability was assessed by Annexin-V-FITC (AnV) and propidium iodide (PI) staining and flow cytometry. Phosphatidylserine externalisation is an early event in programmed cell death and can be detected by AnV binding. PI dye is excluded from cells with an intact membrane. In late apoptosis or necrosis, propidium iodide uptake occurs as membrane integrity is lost. AnV and PI staining can therefore differentiate between viable cells (AnV^−ve^/PI^−ve^) and those undergoing early (AnV^+ve^/PI^−ve^), late apoptotic (AnV^+ve^/PI^+ve^) or necrotic cell death (AnV^−ve^/PI^+ve^) (Fig. 2A)^24^.

After 4 hours, *in vitro*, 93.45 ± 0.74% of neutrophils were AnV^−ve^/PI^−ve^ (see Supplementary Figure 2) and a small proportion of neutrophils were AnV^+ve^/PI^−ve^ (2.05 ± 0.59%) or AnV^+ve^/PI^+ve^ (2.52 ± 0.67%) (Fig. 2A,B). AgNP or Ag Bulk particles have little impact on neutrophil viability at 4 hours and the AnV/PI staining profile was comparable to untreated control neutrophils (Fig. 2B). Untreated neutrophil viability significantly decreased after 20 hours *in vitro*; 43.91 ± 15.17% of neutrophils were AnV^−ve^/PI^−ve^ and 41.26 ± 18.04% of neutrophils were AnV^+ve^/PI^−ve^, representing a significant increase in early apoptosis from 4 hours (Fig. 2C, p = 0.015). A small proportion of untreated neutrophils were also AnV^+ve^/PI^+ve^ (Fig. 2C, 8.52 ± 3.9%), indicative of late stage apoptosis or secondary necrosis^24^. As neutrophils have an estimated half-life of ~14 hours^11^, this decrease reflects natural attrition, *in vitro*.

AgNP or Ag Bulk particles had little impact on total neutrophil viability at 20 hours, and the AnV/PI staining profiles were comparable to controls. However, whilst the total number of apoptotic cells was unchanged, AgNP altered the percentage of neutrophils in the different stages of apoptosis (Fig. 2C). AgNP decreased the percentage of AnV^+ve^/PI^−ve^, early apoptotic neutrophils from 41.26 ± 18.04% to 27.3 ± 7.54% (Fig. 2C, p = 0.1270), and increased the percentage AnV^+ve^/PI^+ve^, late apoptotic neutrophils from 8.52 ± 3.9% to 31 ± 3.67% (Fig. 2C, p = 0.0011); the percentage of AnV^−ve^/PI^+ve^, necrotic neutrophils remained unchanged (6.32 vs 6.18%). This data extends the observation that AgNP cause apoptotic, rather than necrotic, death^25^. As double labelling our cells with AnV and PI enables further differentiation between the stages of apoptotic cell death, our data shows silver nanoparticles may enhance neutrophil transit through apoptosis to late apoptosis.

### AgNP triggers activation of mature circulating neutrophils

Expression of cell surface markers can be used to track neutrophil activation^8,15^. We investigated whether the AgNP dependant change in neutrophil morphology (Fig. 1) associated with altered CD62L (L-selectin), CD16 (IgG Fc γRIII receptor) and CD11b (B2 integrin) expression. CD16 expression aids the distinction of neutrophils from other granulocytes, such as eosinophils, and CD16 expression in mature neutrophils is increased^9,14,26^. Neutrophil CD16 expression is also a marker of apoptotic status^27^ and CD16 and CD62L expression is markedly reduced in aged and apoptotic neutrophils^28^.

**Figure 3:**
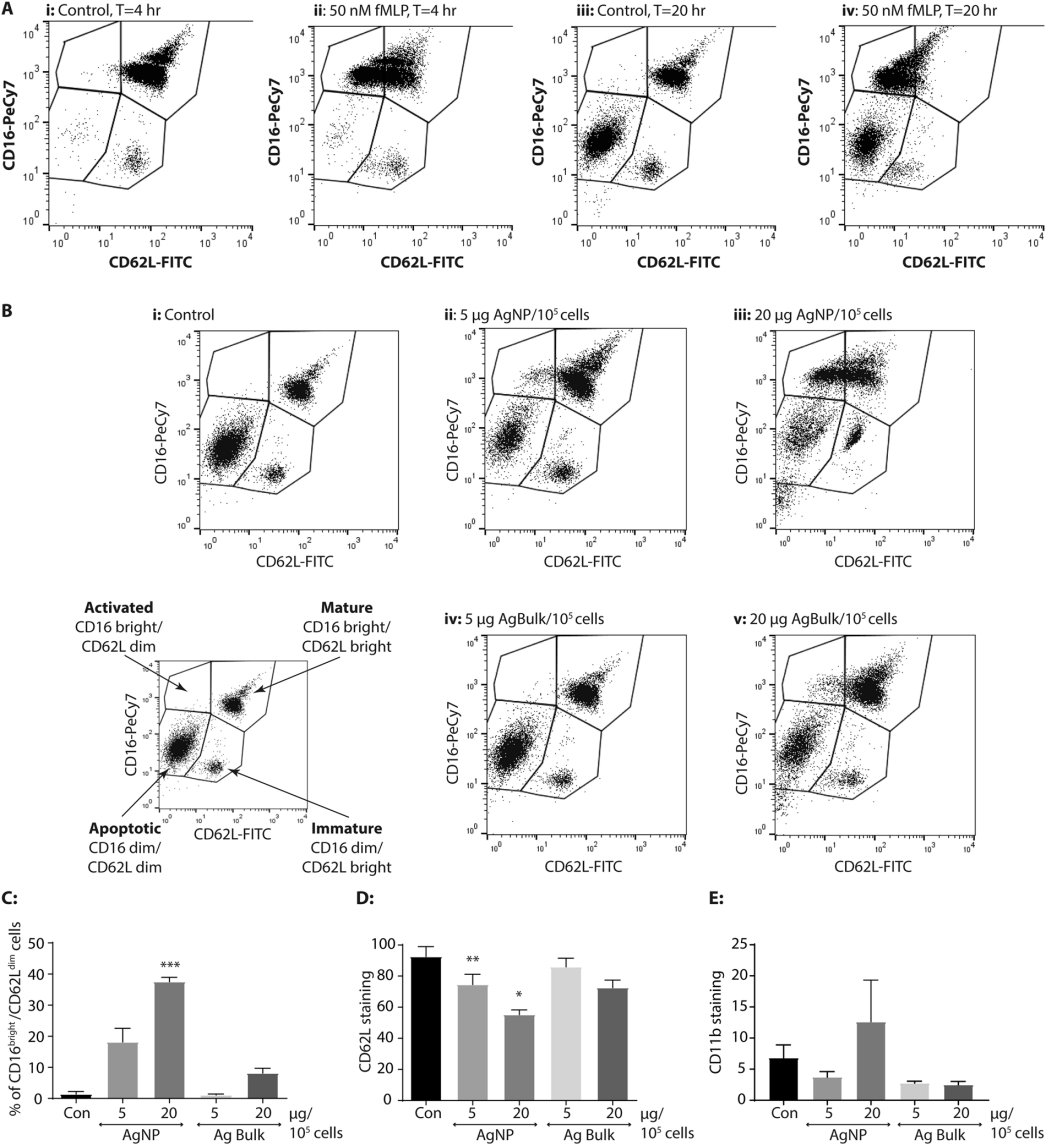
Exposure to silver nanoparticles triggers neutrophil activation. (**A**) Representative profiles obtained from a typical flow cytometry analysis of CD16-PeCy7 and CD62L-FITC stained control (i and iii) or 50 nM fMLP (ii and iv; n = 2) treated human neutrophils, after 4 hr (panel i and ii) or 20 hr (panel iii and iv) *in vitro*. (**B**) Representative profiles obtained from a typical flow cytometry analysis of human neutrophils 20 hr after treatment with silver nanoparticles, staining with CD16-PeCy7 and CD62L-FITC. Panel i: control, ii and iii: 5 or 20 µg AgNP; iv and v: 5 or 20 µg Ag Bulk particles per 10^5^ cells; (**B**) Inset panel: division of neutrophil populations based on CD62L-FITC and CD16-PeCy7 staining profile. Sub-populations were identified by further gating on their CD16 status and the percentage of CD16^bright^/CD62L^dim^ cells (**C**), geometric mean of CD62L staining of CD16^bright^ cells (**D**) and geometric mean of CD11b staining of CD62^bright^/CD16^dim^ cells (**E**) was analysed. Data is expressed as the mean ± SEM where n = 4. *p < 0.05 **p < 0.01 ***p < 0.001.

CD16 and CD62L staining showed the majority of neutrophils within the control population at 4 hr were mature CD16^bright^/CD62L^bright^ (Fig. 3A panel i). A distinct population of CD16^dim^/CD62L^bright^ cells were also evident in the isolated neutrophil population at 4 hrs (Fig. 3A panel i), thought to represent immature circulating neutrophils^29^. Control neutrophils displayed intense CD62L staining and rapid shedding of CD62L in response to treatment with the known neutrophil activator formyl-Met-Leu-Phe (fMLP) (Fig. 3A). These findings show the neutrophils used in this study were not activated by the isolation process, but were highly responsive to activation in the culture conditions used here (Fig. 3A panel ii and iv).

By 20 hr in culture, three major populations of CD16 and CD62L stained neutrophils were evident; CD16^bright^/CD62L^bright^, CD16^dim^/CD62L^bright^ and CD16^dim^/CD62L^dim^ (Fig. 3A, panel iii). In keeping with the viability data presented in Fig. 2, approximately 60% of neutrophils were CD16^dim^/CD62L^dim^ at 20 hours, indicative of mature, apoptotic neutrophils, whilst 39.48 ± 4.25% of the total neutrophil population were CD16^bright^, indicative of non-apoptotic cells (Fig. 3A,B). Untreated CD16^bright^ neutrophils displayed high CD62L staining (Fig. 3B, panel i; 3D, 92.57 ± 6.43), whilst little CD62L staining was evident on CD16^dim^ neutrophils (Fig. 3B, panel i; Fig. 3D), indicating non-apoptotic, control neutrophils were not activated, even after 20 hours in culture.

AgNP exposure dramatically decreased CD62L expression in the CD16^bright^ population, promoting a concentration dependant increase in prevalence of CD16^bright^/CD62L^dim^ neutrophils (Fig. 3B panel ii and iii vs i; Fig. 3C, p < 0.0003) and significantly decreasing CD62L staining in CD16^bright^ population (Fig. 3D, p = 0.024). AgNP treatment also caused CD16^bright^ neutrophils to increase CD11b expression and CD11b staining increased from 6.82 ± 2.08 to 12.61 ± 6.71 (Fig. 3E). Ag Bulk had little impact on the CD62L or CD11b status of CD16^bright^ cells (Fig. 3C,E). Higher concentrations of Ag Bulk caused a small increase in the number of CD16^bright^/CD62L^dim^ neutrophils (Fig. 3C, p = 0.0525) and a small decrease in CD62L staining (Fig. 3D, p = 0.0784) however neither were significant. CD11b staining of CD16^bright^/CD62L^bright^ neutrophils was unchanged in the presence of Ag Bulk (Fig. 3E). Together, this suggests AgNP trigger activation of mature, circulating neutrophils.

### Exposure to AgNP may trigger maturation of immature human neutrophils

**Figure 4:**
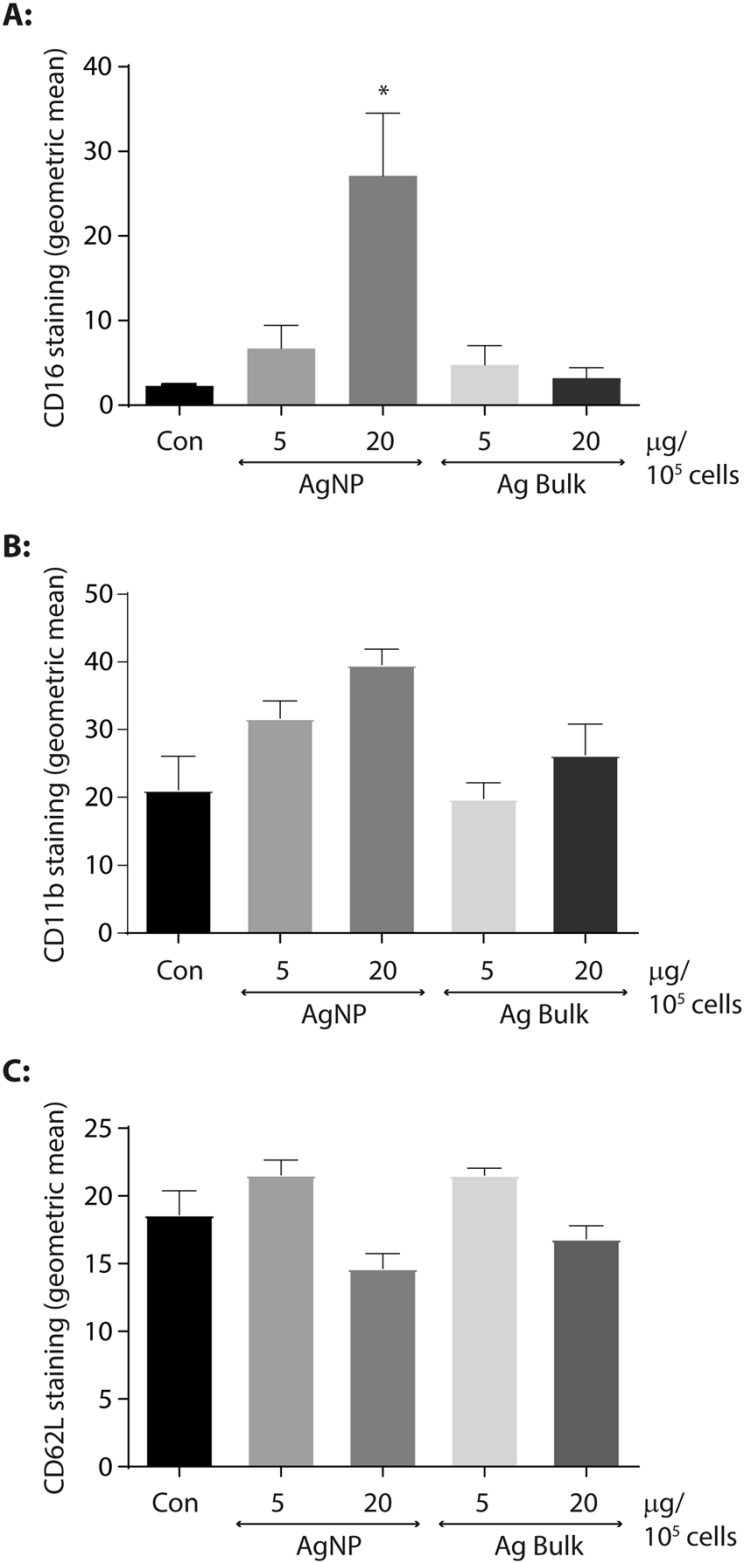
Exposure to silver nanoparticles triggers neutrophil maturation. The geometric mean of CD16-Pe-Cy7 (**A**) CD11b-APC (**B**) and CD62L-FITC (**C**) staining was determined in the CD16^dim^/CD62L^bright^ sub-population of untreated human neutrophils (Con) or following treatment with 5 or 20 µg AgNP or Ag Bulk particles per 10^5^ cells for 20 hr at 37 °C. Data is presented as the geometric mean ± SEM, where n = 3; *p < 0.05.

Two distinct CD16^dim^ populations were evident in our isolated neutrophils depending on their CD62L status (Fig. 3A) and at t = 20 hrs CD16^dim^/CD62L^dim^ and CD16^dim^/CD62L^bright^ populations represented 57.3 ± 4.38% and 5.28 ± 0.69% of the overall neutrophil pool, respectively (Fig. 3A,B). In control neutrophils, the major population of CD16^dim^/CD62L^dim^ neutrophils were also CD11b^dim^ (Fig. 3E). Curiously, the CD11b staining intensity of control CD16^dim^/CD62L^bright^ neutrophils was significantly greater than CD11b staining of CD16^bright^/CD62L^dim^ cells (21.11 ± 4.94% vs 4.76 ± 0.44%; p = 0.0384; compare the control in Fig. 4B with Fig. 3E).

Subtle changes in receptor expression were also observed in the CD16^dim^ population following treatment with AgNP. AgNP exposure dramatically altered the staining profile of the CD16^dim^/CD62L^bright^ population (Fig. 3B, panels ii and iii vs iv and v), significantly increasing CD16 staining from 2.28 ± 0.18 to 27.13 ± 7.39 (Fig. 4A, p = 0.023), and causing a modest increase in CD11b expression from 21.11 ± 4.94 to 39.62 ± 2.29 (Fig. 4B). CD16 and CD11b staining of the CD16^dim^/CD62L^bright^ population was unaffected by Ag Bulk exposure (Fig. 4A,B) or indeed treatment with fMLP which triggered CD62L shedding in this population rather than an increase in CD16 expression (Fig. 3A, compare panel iv with iii).

By contrast, CD62L staining in the CD16^dim^/CD62L^bright^ population was relatively unaffected by AgNP or Ag Bulk exposure (Fig. 4C). As CD16 expression increases in mature neutrophils^9,29^, this data suggests AgNP exposure may trigger maturation, yet not activation, of circulating immature, human neutrophils.

### Neutrophil activation is accompanied by cytokine release

**Figure 5:**
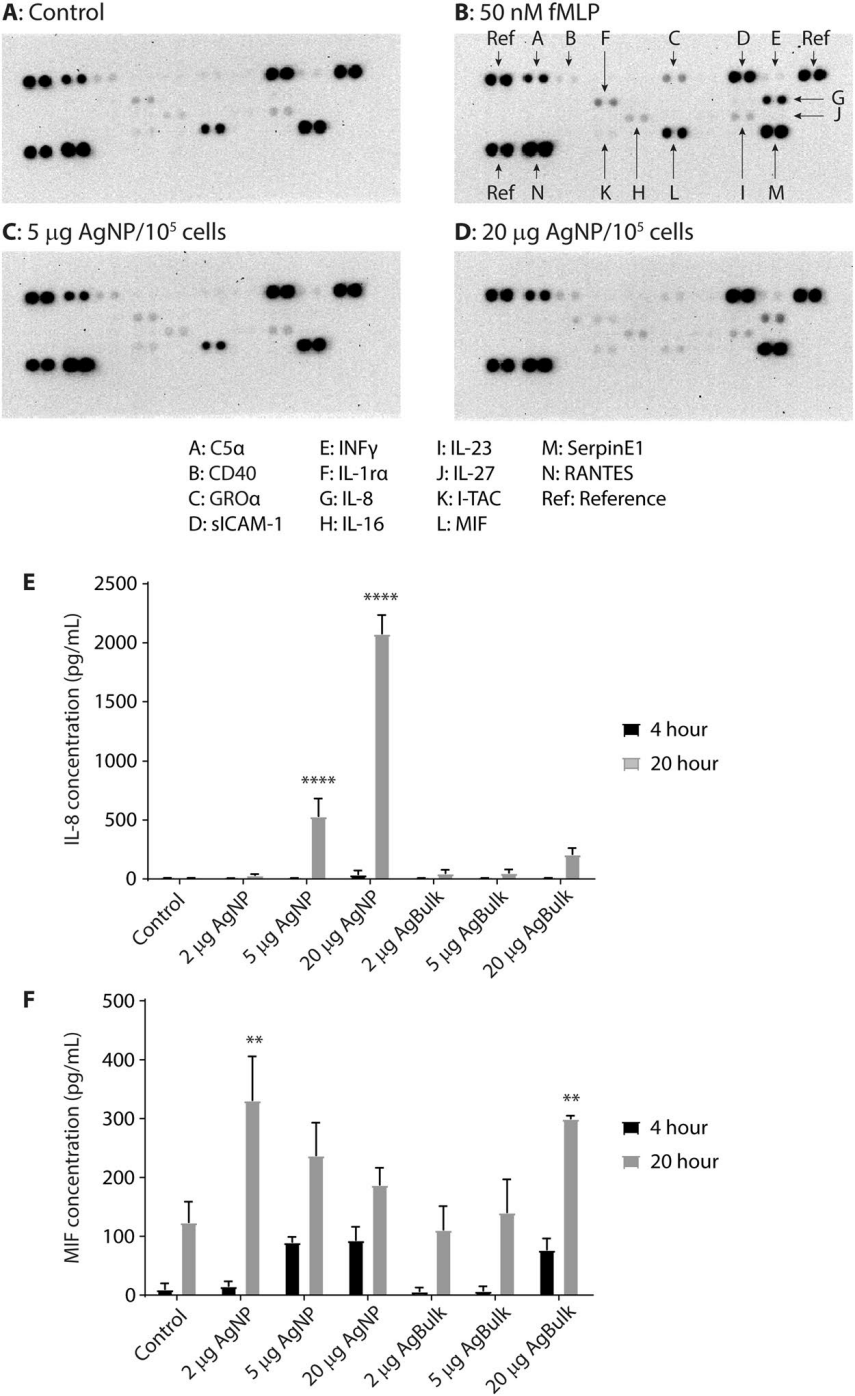
The profile of cytokines released from human neutrophils following exposure to silver nanoparticles. The presence of cytokines in the culture medium from control, untreated neutrophils (**A**) or those exposed to 50 nM formyl-Met-Leu-Phe (fMLP, **B**), 5 µg (**C**) or 20 µg (**D**) AgNP per 10^5^ cells for 4 hr at 37 °C was assessed using a cytokine profiler array. Duplicate spots of interest are numbered and their identity is shown in the key below. The concentration of IL-8 (**E**) or MIF (**F**) released into the culture media from untreated neutrophils (Con) or following exposure to 2, 5 or 20 µg AgNP or AgBulk particles per 10^5^ cells for 4 or 20 hr at 37 °C was further assessed by ELISA. Data is expressed as the mean cytokine concentration ± SEM, where n = 4; **p < 0.01; ***p < 0.001.

Neutrophil activation is associated with degranulation and release of inflammatory mediators^30^. The profile of cytokines released from AgNP treated neutrophils was investigated via profiler array (Fig. 5). Four hour incubation was chosen as it correlates with a change in neutrophil granularity (Fig. 1B) without any change in viability (Fig. 2), representing an early time point where neutrophils are responding to AgNP and may release specific mediators to modulate the immune system to clear the particle burden.

**Figure 6:**
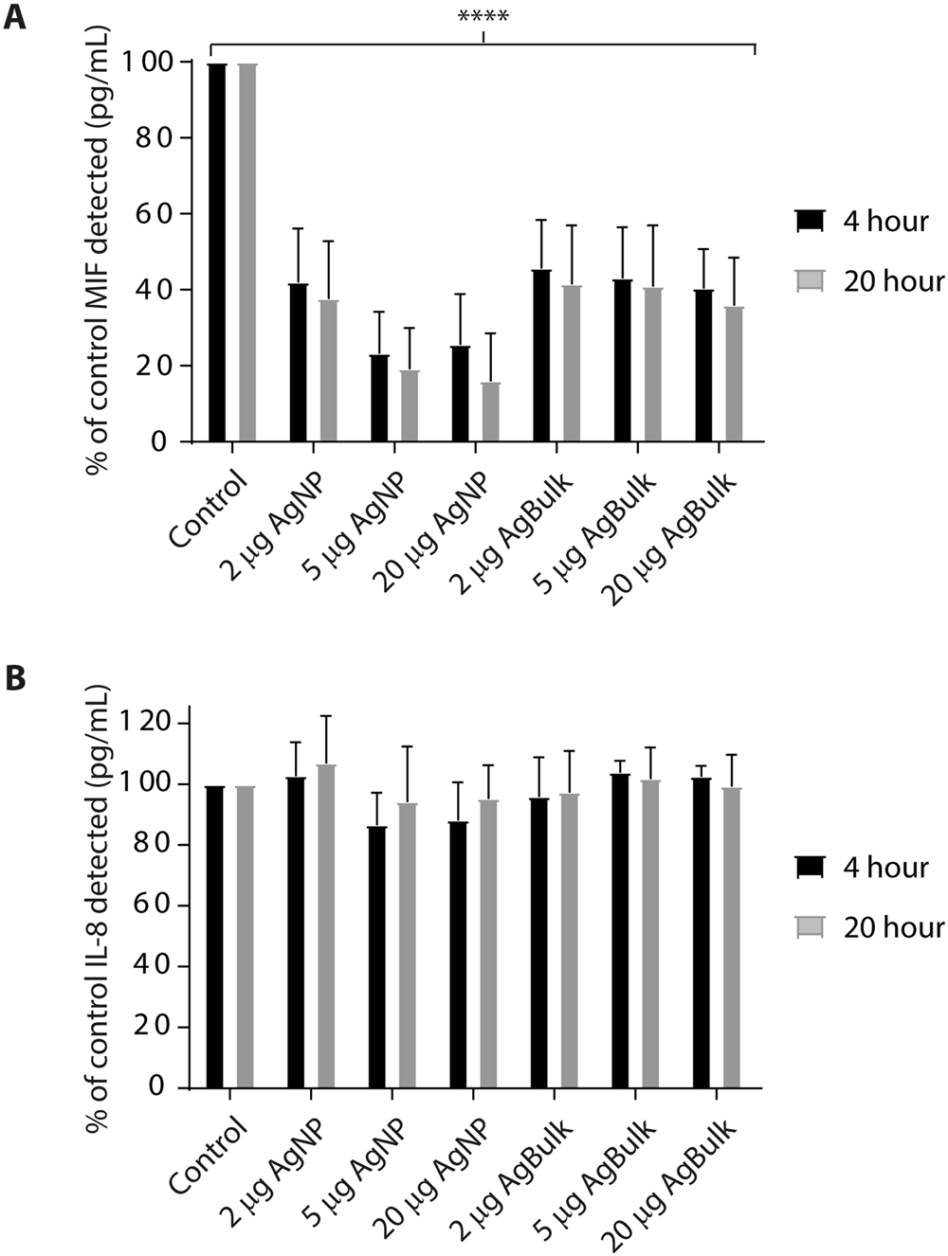
The impact of silver nanoparticles on cytokine detection. Recombinant MIF (**A**) or IL-8 (**B**) (250 pg/ml) was incubated without (Control) or with 2, 5 or 20 µg AgNP or Ag Bulk particles per 10^5^ cells in culture medium containing autologous serum for 4 or 20 hr at 37 °C. The concentration of MIF or IL-8 in the clarified culture medium was assessed via ELISA. Data is expressed as the percentage of control cytokine concentration ± SEM, n = 3; *p < 0.05; ***p < 0.001.

Several cytokines and chemokines were detected in the culture medium from untreated neutrophils, including macrophage inhibitory factor (MIF), soluble intracellular adhesion molecule (sICAM), RANTES (regulated on activation, normal T cell expressed and secreted; CCL5) and Serpin1 (Fig. 5A; Supplementary Figure 1). fMLP was included as a positive control to trigger neutrophil activation and caused a 12 fold increase in interleukin-8 (IL-8) release (Fig. 5B, spot G) and a ~1.5 fold increase in growth regulated protein alpha (GROα) and IL-1 receptor antagonist (IL-1ra) release (Fig. 5B, spots C and F respectively). Exposure to 5 and 20 µg AgNP/10^5^ cells caused measurable increases in selected cytokines (Fig. 5C and D); IL-8 was increased 8 fold by 20 µg AgNP/10^5^ cells whilst IL-16 and IL-27 were increased 1.75 and 2.4 fold respectively (Fig. 6, A vs C and D, spots H and J respectively). GROα, IL-1ra, IL-6 or IL-17 release was unaffected by the presence of AgNP (Fig. 5C,D). Surprisingly, AgNP caused a notable decrease in the MIF concentration in the culture medium (Fig. 5C and D vs A; spot L); 5 and 20 µg AgNP/10^5^ cells decreased MIF by 20% and 86% whilst fMLP had no effect on the MIF concentration (Fig. 6B, spot L). This shows key cytokine release accompanies neutrophil activation in response to AgNP.

### Silver nanoparticles enhance the release of pro-inflammatory cytokines

Having identified MIF and IL-8 as key cytokines released from AgNP activated neutrophils, their concentration in the culture medium was validated and quantified at early and later time points following neutrophil exposure to AgNP or Ag Bulk by ELISA.

IL-8 was barely detectable in the culture medium of untreated neutrophils at 4 and 20 hours (Fig. 5E, ~5 pg/mL). AgNP caused a small, yet insignificant increase in IL-8 release at 4 hours and a dramatic increase in IL-8 release at 20 hours (Fig. 5E, 2077 ± 157 pg/mL, p < 0.0001). Ag Bulk had little impact on IL-8 release; a small yet insignificant increase in IL-8 release was detected at 20 hours after exposure to 20 µg Ag Bulk/10^5^ cells (210.3 ± 52 pg/mL, Fig. 5E, p = 0.11).

Low concentrations of MIF were detected in the culture medium of untreated neutrophils at 4 and 20 hour (16.5 ± 9 pg/mL and 107.9 ± 29 pg/mL respectively, Fig. 5F). AgNP triggered further MIF release at 4 hours and MIF concentrations were 6.3 fold greater than untreated controls in the presence of 20 µg AgNP/10^5^ cells (Fig. 5F). MIF was also elevated by AgNP at 20 hours, however lower concentrations of AgNP had a greater impact on MIF release; MIF was 4.3, 2.7 and 1.9 fold greater than control cells in the presence of 2, 5 and 20 µg AgNP/10^5^ cells, respectively (Fig. 5F, p = 0.0017).

Low concentrations of Ag Bulk had little effect on MIF concentrations at 4 or 20 hours (Fig. 5F), however 20 µg Ag Bulk/10^5^ cells increased MIF release at both 4 and 20 hours (Fig. 5F, p = 0.0081). This data suggests cytokine release accompanies neutrophil activation by AgNP.

### Silver particles interfere with quantification of MIF detection

Nanoparticles can interact with and adhere to proteins, creating a protein corona^31^. Such particle:protein interactions may mask epitope binding sites and impact upon protein detection. The observed reduction in MIF release from neutrophils exposed to higher concentrations of AgNP (Fig. 5F) was surprising and we were curious as to whether this represented interference created by MIF:silver particle interactions which may reduce MIF detection. To investigate this, 250 pg/mL recombinant MIF was incubated with AgNP or Ag Bulk in culture medium alone for 4 and 20 hours then quantified via ELISA. Little, if any change, in MIF concentration was observed in the culture medium only, indicating MIF is stable at 37 °C (Fig. 6A). AgNP or Ag bulk particles dramatically reduced MIF concentrations at 4 and 20 hours to ~60% of control values (Fig. 6A, p < 0.0001). MIF detection was further reduced by increased AgNP particle concentration (Fig. 6A, p = 0.046); increased Ag Bulk concentration had little effect on MIF detection (Fig. 6A).

The impact of particles on IL-8 quantification was also assessed. IL-8 detection was unaffected by AgNP or Ag Bulk and no significant difference in IL-8 concentration was detected 4 or 20 hours after exposure to particles (Fig. 6B). This suggests interference occurs between AgNP, Ag Bulk and MIF which dramatically reduces MIF estimation and this is specific to MIF, as IL-8 detection is unaffected. The reduction in MIF concentration observed following treatment of neutrophils with AgNP (Fig. 5) may therefore reflect particle interaction and interference with the assay, rather than reduced MIF release.

## Discussion

Peripheral blood contains a heterogeneous population of circulating neutrophils at various stages of maturity and mobility^7^. A growing body of literature also supports the presence of neutrophil subpopulations with differing inflammatory phenotypes and ability to modulate adaptive immune responses, in particular T-cell function^17^. Three distinct neutrophil sub populations were identified in acute systemic inflammation based on their CD16/CD62L staining profile^15^, including phenotypically mature CD16^bright^/CD62L^bright^, immature, CD16^dim^/CD62L^bright^ neutrophils, and a distinct CD16^bright^/CD62L^dim^ sub population that could potently suppress T-cell proliferation *in vitro*^15^. These immunosuppressive CD16^bright^/CD62L^dim^ neutrophils displayed reduced phagocytic index and reactive oxygen species production compared to classic pro-inflammatory, CD16^bright^/CD62L^bright^ neutrophils^16^. Circulating high and low density neutrophils have also been identified, with low density neutrophils predominating in inflammation and cancer patients^32^. Circulating immunosuppressive immature neutrophils (granulocytic myeloid-derived suppressor cells, G-MDSC) can directly suppress CD8^+^ T-cells proliferation and modulate the immune synapse via arginase and ROS production^17^ indicating the immunosuppressive capacity of certain circulating neutrophil sub-populations. Neutrophils are highly responsive to environmental ques (reviewed by^7^) and neutrophil polarisation from classic pro-inflammatory, N1 phenotype, to more pro-angiogenic, pro-tumourigenic N2 phenotype in response to TGF-β is observed in tumours^33^. These findings highlight the functional plasticity and phenotypic diversity within circulating neutrophil population and challenge the traditional view of neutrophils as a homogenous population of solely pro-inflammatory cells.

The majority of circulating neutrophils isolated from our healthy donors were mature, CD16^bright^/CD62L^bright^ neutrophils and circulating CD16^bright^/CD62L^dim^ neutrophils were not identified (Fig. 3A). Silver nanoparticles triggered CD62L shedding from the CD16^bright^/CD62L^bright^ sub-population, and a noticeable increase in the prevalence of immunosuppressive, CD16^bright^/CD62L^dim^ neutrophils. As CD62L expression is indicative of activation status^13^, our findings show nano-scaled silver activates circulating neutrophils; supported by the dramatic increase in IL-8 release from neutrophils exposed to nanoparticles. Neutrophils release IL-8 in response to a variety of stimuli. As a potent chemoattractant, it promotes recruitment of circulating neutrophils to sites of infection, their activation and degranulation^4^. Elevated IL-8 indicates neutrophils respond to silver nanoparticles, *in vitro*, and may signal to activate neighbouring neutrophils to aid particle clearance. Silver nanoparticles are rapidly internalised by neutrophils^34^, and this correlates with increased neutrophil size^25^. Silver nanoparticles increased both size and granularity of our neutrophil populations suggesting nanoparticle internalisation is the trigger for neutrophil activation.

Activated neutrophils are a marker of inflammation, however this does not always equate with a damaging, pro-inflammatory immune response as neutrophils can also control and resolve inflammation by clearing inflammatory stimuli^35^. Activated neutrophils release batteries of inflammatory and immunomodulatory cytokines and chemokines, including potent pro-inflammatory cytokines, TNFα (tumour necrosis factor alpha), INFγ and IL-17^36^. In this study, neutrophil activation was not accompanied by pro-inflammatory cytokine release and INFγ (interferon gamma), IL-1ra, IL-17 or Groα were absent from the culture medium of nanoparticle treated neutrophils. Nano-scaled silver also triggered the emergence of a CD16^bright^/CD62L^dim^ population of neutrophils that have been previously suggested to inhibit T-cell responses^15^, suggesting silver nanoparticles promote resolution of inflammation. Neutrophil activation here may therefore stimulate particle clearance without promoting a damaging inflammatory response.

Neutrophil death can influence and direct immune responses^37^. Primary necrosis was not observed in silver treated neutrophil preparations, in keeping with the absence of a damaging inflammatory response to silver nanoparticles. However increased late apoptotic neutrophil death was observed at 20 hours following silver nanoparticle exposure. Apoptotic neutrophils are cleared, *in vivo*, by macrophage efferocytosis^38^. The absence of macrophages, *in vitro*, means apoptotic neutrophils are not effectively cleared and therefore progress to late apoptosis or secondary necrosis. Efferocytosis of apoptotic neutrophils switches macrophage function to an anti-inflammatory phenotype, and ultimately resolution of tissue inflammation^39^. Neutrophil apoptosis is therefore considered anti-inflammatory^40,41^ whilst necrotic neutrophil death, and leakage of neutrophil’s cellular contents, is pro-inflammatory and linked to tissue damage^42^. Soares *et al*., found PVP coated silver nanoparticles induced necrotic neutrophil cell death, yet did not determine whether this was primary or secondary necrosis^34^. Our data is in keeping with others, who show silver nanoparticles induce a small increase in apoptotic neutrophil cell death^25,43^, and supports our observation that silver nanoparticles promote an anti-inflammatory, rather than pro-inflammatory, response.

MIF is a pleotropic cytokine released in response to pro-inflammatory cytokines and is involved in inflammatory pathogenesis^44^. Its presence in the media of silver treated nanoparticles is at odds with the emergence of the anti-inflammatory neutrophil sub-population and absence of other pro-inflammatory cytokines. MIF is passively released from human neutrophils undergoing secondary necrosis, due to reduced membrane integrity^45,46^. In the absence of macrophages, increased MIF release may reflect an *in vitro* phenomenon resulting from lack of neutrophil clearance, rather than silver nanoparticle dependant release of this pro-inflammatory cytokine^47^.

Protein adhesion onto silver nanoparticles was clearly evident in the presence of MIF, and nano and bulk silver particles interfere with MIF quantification. Curiously IL-8 was unaffected by particles and the IL-8 concentrations obtained were as expected, suggesting certain cytokines are more affected by nanoparticle adhesion than others. Particles may therefore cause underestimation of selected cytokine concentrations in the culture medium or biological fluids surrounding inflammatory cells. As adsorption onto particles can alter the conformation and function of coronal proteins^48^ and attenuate degranulation enzyme activity^49^, it is possible particle adhesion and interference may impact upon cytokine function. The reduced MIF concentrations observed in silver particles preparations may therefore be due to MIF denaturation preventing antibody recognition. MIF adsorption in the presence of silver particles may also compromise MIF functionality and potentially negate its inflammatory effects. Further analysis of the functionality of MIF released from silver particle treated neutrophils is required to confirm this.

Our model permits simultaneous investigation of the silver nanoparticle’s effects on circulating mature, CD16^bright^/CD62L^bright^ and immature, CD16^dim^/CD62L^bright^ neutrophils. Silver nanoparticles triggered CD62L shedding and activation of mature circulating neutrophils however CD62L shedding and activation was not observed in immature neutrophil population. Instead, silver nanoparticles dramatically increased CD16 expression in the immature neutrophils. As CD16 is a marker of neutrophil maturation^9^, this suggests nano-silver could trigger maturation of circulating CD16^dim^/CD62L^bright^ neutrophils. Curiously, immature neutrophils displayed higher basal CD11b staining compared to mature neutrophils and a trend for increased CD11b expression following AgNP treatment. As CD11b is an adhesion molecule and increased expression is indicative of activation, this observation suggests immature cells may be in a more “primed” yet differential state of activation compared to mature neutrophils. These findings underscore the heterogeneity within circulating neutrophils and their differential responsiveness to environmental cues, such as nanoparticles.

CD16 enables neutrophil recognition of opsonising antibodies, facilitating phagocytosis and destruction of pathogens^50^. Silver has antimicrobial properties, inhibiting bacterial respiratory chain enzymes^51^ and disrupting the structure/function of bacterial membrane proteins and lipids^52^. Silver’s ability to promote maturation of the CD16^dim^/CD62L^bright^ population, and their resultant increase in CD16 expression, could therefore enhance silver’s bactericidal activity by promoting internalisation, and clearance of microbes by neutrophils. Isolation, and further analysis of this sub-population is required to confirm this.

Our analysis brings clarity to recently published data on silver nanoparticle’s impact on neutrophil function and the conclusions drawn regarding silver particles’ impact on neutrophil viability and resultant pro-inflammatory status. We show silver nanoparticle dependant activation of human neutrophils is accompanied by limited cytokine release, that silver particles promote apoptotic, rather than tissue damaging, pro-inflammatory, necrotic cell death, and that silver nanoparticles differentially effect circulating sub-populations of neutrophils, promoting the emergence of a subpopulation which has previously been suggested to have immunosuppressive characteristics^15^. By analysing these key endpoints, we show silver nanoparticles significantly impact upon the maturity and activation status of circulating human neutrophils and may promote resolution of inflammation by increasing the prevalence of immunosuppressive neutrophils. We therefore propose that neutrophil’s response to silver nanoparticles, even at relatively high particle concentrations, is a controlled, inflammatory response to clear and resolve particle-induced inflammation. Bulk silver particles had no notable effect on the activation or maturation status of human neutrophils, indicating that the effects are specific to nano-sized particles. Whilst our analyses bring a degree of clarity to the single cell neutrophil model, a detailed functional analysis of the individual neutrophil subpopulations, their ROS production and degranulation will form part of our future work to assess the potential immunosuppressive properties of neutrophil subsets. Further analyses are also required to understand the human macrophage response to particles and particle-activated neutrophils, to appreciate the interdependent nature of the immune system.

*In vitro* interactions between human neutrophils and silver particles have previously been investigated^25,34,43^, however differing conclusions have been drawn on silver nanoparticle’s impact on neutrophil viability, and the particle concentrations required to trigger an effect, or even toxicity. Variation in experimental design exists in nanotoxicological studies, regarding particle concentration, cell density, plate size and culture volume used, making direct comparison of *in vitro* data problematic. As neutrophils grow as a suspension, differences in number of cells/mL and the resultant cell:particles ratio may have a significant effect on cellular interactions. Our data is presented as the concentration of particles/number of cells to enable comparability with different studies and experimental set-ups. Particle concentrations used here are comparable to those used by others (see Supplementary Table 1)^34,43^.

Variation also exists in particle preparations and *in vitro* culture conditions used, with researchers employing buffered solutions rather than serum to prepare particles^25^ or foetal calf serum instead of autologous serum to supplement growth media^34^. Nanoparticles adsorb biological molecules forming a protein corona^53^. This can significantly alter nanoparticle’s properties and their interaction with cell surface receptors, influencing cellular recognition of particles, their uptake and potential toxicity^54^. The absence of a corona in particles prepared in protein free, buffered solutions, or the presence of bovine forms of biological proteins, may significantly alter a nanoparticle’s physiochemical properties and account for differences in silver nanoparticle toxicity and neutrophil viability observed by others^25,34^. Donor matched autologous serum was utilised here, not only to provide the correct support for the primary cells, but to simulate the human protein corona that would form around nanoparticles *in vivo*^31^, and more accurately reflect possible particle-corona effects on neutrophil-particle interactions.

Our model engages with the heterogeneous nature of circulating neutrophil populations and the importance of biological protein corona, making it a powerful model to study the impact of silver nanomaterials. Circulating neutrophils from healthy adults were used in this study, however neutrophil sub-populations can differ with age, and the percentage of CD16^bright^/CD62L^dim^ neutrophils increases in elderly individuals^16^. Neutrophil exposure to ultra-high molecular weight polyethylene can prevent neutrophil responses to *Staphlococcus aureus*^55^ suggesting other nanomaterials may have differing effects on aged or immunocompromised individuals’ neutrophil function. Analysing the nuances of selected circulating neutrophil sub-populations and the impact of silver particles in, *in vitro* neutrophil toxicological models is essential for the safe implication of silver nanoparticles in biotechnology and biomedical devices.

As nanoparticles quickly become an established technology, their production and use will only increase. This has worldwide implications for industry wishing to trade in countries covered by REACH legislation, and risk assessors and regulators that need to balance hazard and risk assessment with the principles of 3 R’s. This will be confounded by a plethora of nanoforms, with divergent characteristics entering the market, that will continue to challenge predictive models, be that *in silico* assessment, or categorisation based on physiochemical characteristics. It is therefore essential we use the most appropriate and sensitive *in vitro* models to inform such predictive tools and hazard assessments, to more accurately predict *in vivo* immune responses following nanomaterial exposure, and thus make informed decisions that move beyond current cell based assumptions.

## Materials and Methods

### Participants

All protocols were ethically approved by the Edinburgh Napier University, Research Integrity Committee and all methods were performed in accordance with the relevant guidelines and regulations. Peripheral venous blood was obtained from healthy volunteers after obtaining signed, informed consent. Twelve healthy male and female participants were used in this study.

### Isolation of polymorphonuclear cells from whole blood using Dextran–Percoll Gradients

Peripheral blood polymorphonuclear cells were isolated by modified dextran–Percoll gradient as previously described^56^. Briefly, sodium citrate solution (final concentration of 0.38%), was added to whole blood to prevent coagulation. Samples were centrifuged at 320 × g for 20 min and the upper platelet rich layer was removed and retained. Dextran (500,000 MW) was added (final concentration of 1.5%) to the pelleted cells and the volume was adjusted to 50 mL with saline. After gentle inversion, cells were allowed to sediment at room temperature for 30 min. The upper cell suspension was removed and washed in saline before the cell pellet was resuspended in 55% Percoll, in phosphate buffered saline (PBS) lacking magnesium and chloride (Invitrogen). A discontinuous double Percoll gradient, containing equal volumes of 70% and 81% Percoll, was prepared and the cell suspension was layered on top before the gradient was centrifuged at 570 × g for 20 min. Neutrophils were collected from the interface between the 70% and 81% Percoll layers, washed twice in saline and resuspended in RPMI media (Invitrogen). The purity of separation was assessed by haematoxylin and eosin staining of cytospun cell preparations. Efficient neutrophil isolation was confirmed by light microscopy and preparations routinely contained <5% eosinophils and mononuclear cells.

Autologous serum was generated from the platelet rich layer obtained from the initial centrifugation. CaCl_2_ was added to a final concentration of 14 mM and incubated at 37 °C for 1 hr to yield autologous serum. This was heat inactivated for 1 hr at 56 °C and clarified by centrifugation before use.

### Particle preparation

Nano (20 nm; #0478HW) and bulk (1.5–2.5 µm; #0472DFS3) silver (Ag) particles were obtained from NanoAmor (Houston, TX, USA). Silver particles were characterised by dynamic light scattering (DLS), to draw comparisons with information provided in the commercial data sheet. The average hydrodynamic diameter (Z-average) was determined using a Zetasizer NanoZS (Malvern Instruments, UK) following the manufacturer’s instructions. Particles (0.1–1 mg/mL) were prepared in ultrapure distilled water and sonicated in a sonicating water bath for 10 min prior to use. Particles were transferred to a disposable cuvette and readings, comprised of data from 15 runs were taken before the mean particle diameter ± SEM of triplicate readings was calculated. The Z-average of 20 nm AgNP and 1.5–2.5 µm Ag bulk particles were 322 ± 13.1 and 1678.3 ± 322.6 d.nm respectively.

For particle preparations for cells, stock solutions of particles were prepared in using RPMI containing 10% heat inactivated autologous serum and sonicated as above. Dilutions of particles were prepared in RPMI plus 10% autologous serum.

### PMNC culture and treatment

For flow cytometry and analysis of cytokine release, neutrophils were seeded in 6 well plates at a density of 5 × 10^6^ cells per well in 2 mL RPMI plus 10% autologous serum. Cells were allowed to rest for 1 hr at 37 °C in a humidified incubator before 1 mL of particle suspension was added, giving a final volume of 3 mL and a final concentration of 2, 5 or 20 µg particles/10^5^ cells (see Supplementary Table 1). In certain situations, neutrophils were also treated with *N-*formyl-methionyl-leucyl-phenylalanine (formyl-Met-Leu-Phe; fMLP) (Sigma) to a final concentration of 50 nM, as a positive control to activate neutrophils. Samples were incubated for 4 or 20 hr at 37 °C before they were harvested and analysed.

### Flow cytometry analysis

All reagents were purchased from BD Biosciences unless otherwise stated. Neutrophils were harvesting by scraping and gentle centrifugation at 100xg at room temperature. Culture media was removed and stored at −80 °C for further analysis via enzyme-linked immunosorbent assay (ELISA). The cell pellet was washed once in ice cold PBS.

For cell surface marker analysis, non-specific binding was blocked by incubating neutrophils with 10% normal mouse serum (Sigma) in PBS for 30 min on ice. Cells were divided into two pools and incubated with fluorescently conjugated mouse monoclonal antibodies towards CD11b, CD62L and CD16. Twenty microliters of FITC CD62L (cat no. 555543) and APC CD11b (cat no. 550019) or 5 µL of PE-Cy7 CD16 (cat no. 557744) or the corresponding volume of isotype matched control antibodies (IgG1κ FITC, cat no. 555748; IgG1κ APC 555751 or IgG1κ PE-Cy7: 557872) were added per 1 × 10^6^ cells and incubated on ice, protected from light, for 30 min. Cells were collected by gentle centrifugation and washed three times in PBS before they were resupended in 0.25 mL cold PBS and analysed by flow cytometry using a Becton Dickson FACSCalibur.

For analysis of cell viability, cells were washed twice with PBS and resuspended at 1 × 10^6^ per mL in Annexin-V binding buffer (Biolegend). Annexin-V FITC (10 µL) and propidium iodide (20 µL) was added and samples were incubated at room temperature in the dark for 15 min; the control was left untreated. Annexin-V buffer (800 µL) was added and samples were analysed by flow cytometry as above. All flow cytometry data was analysed using FlowJo software.

### Cytokine Array

The cytokine content of the culture medium was assessed via Proteome Profiler Human Cytokine Array (Panel A Kit, R&D Systems, Minneapolis, USA). Culture medium from neutrophils treated with or without nanoparticles (0.75 mL) was mixed with 0.75 mL of Array Buffer 4. A cocktail of biotinylated antibodies was added and incubated at room temperature for 1 hr. The sample-antibody mixture was added to the blocked membrane and incubated at 4 °C for 18 hr. Following 3 × 10 min washes, streptavidin-HRP antibody was added at a dilution of 1:2000 and incubated at room temperature for 45 min. The membranes were washed, probed with ECL Western Blotting Substrate (Thermo Scientific) and imaged using a ChemiDoc™ XRS + System (BioRad). The intensity of each spot was measured using the ImageJ software and the average pixel density of each duplicate spot was expressed for comparative analysis.

### Cytokine ELISA

Human MIF and CXCL8/IL-8 were measured by ELISA using DuoSet ELISA kits (R&D systems) according to the manufacturer’s instructions. Briefly, 96 well immunoplates (Nunc) were coated with capture antibody overnight, washed and blocked with 1% bovine serum albumin (BSA) in PBS. One hundred microliters of clarified culture medium was added per well and incubated for 2 hr at room temperature; samples were prepared in triplicate. A standard curve was generated from known standards of IL-8 and MIF. Captured cytokines were detected by biotinylated detection antibody and streptavidin conjugated HRP. After washing, substrate was added and colour was allowed to develop for 20 min at room temperature. The reaction was stopped with 2 N H_2_SO_4_ and absorbance was measured at 540 nm and 570 nm. The concentration of cytokine per sample was calculated using a four parameter logistic curve fit of the standard concentrations.

### Assessing particle interference

MIF and IL-8 standards (R&D systems) were prepared according to the manufacturer’s instructions. Particles were prepared in medium containing 10% autologous serum and sonicated for 10 min prior to dilution to either 0.1, 0.25 or 1 mg/mL. These concentrations of particle preparations are identical to those used to prepare 2, 5 and 20 µg particles/10^5^ cells. One millilitre of particle preparation was added to 2 mL of medium containing 10% autologous serum and MIF or IL-8 was added to a final concentration of 250 pg/mL. Cytokine was also incubated in the absence of particles as a control. Particles and cytokine were incubated at 37 °C for either 4 or 20 hr before the medium was collected and clarified by centrifugation at 3000 rpm. Medium was stored at −80 °C and MIF and IL-8 concentrations were assayed by ELISA.

### Statistical analysis

Statistical analyses were conducted using GraphPad Prism v7.0 (Graphpad software Inc.). One way or two way repeated measures ANOVA with Tukey or Dunnett’s multiple comparison *post-hoc* test were performed and data was deemed significant if p < 0.05.

### Availability of data and material

All data generated or analysed during this study are included in this published article and its supplementary information files.

### Ethics approval and consent to participate

All protocols were ethically approved by the Edinburgh Napier University, Research Integrity Committee and all methods were performed in accordance with the relevant guidelines and regulations. Peripheral venous blood was obtained from healthy volunteers after obtaining signed, informed consent.

## Electronic supplementary material

Supplementary information

Supplementary data file 1
