# Supplementary material for: Silver nanoparticles promote the emergence of heterogeneic human neutrophil sub-populations
Source: Sci Rep. 2018 May 14;8:7506. doi: 10.1038/s41598-018-25854-2 (PMC5951814; doi:10.1038/s41598-018-25854-2)
Supplement: Supplementary file 1 — Supplementary information [file 41598_2018_25854_MOESM1_ESM.pdf]

Silver nanoparticles promote the emergence of heterogeneic human neutrophil sub-populations.

Jennifer A. Fraser[1], Sadie Kemp, Lesley Young, Mark Ross, Morag Prach, Gary R. Hutchison, Eva Malone

School of Applied Sciences, Edinburgh Napier University, Sighthill Campus, Edinburgh, EH11 4BN, UK.

[1]Corresponding author (j.fraser@napier.ac.uk)

Supplementary table 1:

Reference	Cell type	No. of cells	Plate size	Assay volume	Particle conc; particles/well	Final particle conc	Assays used
**This study**	Human neutrophils	5 x10^6 cells/well	6 well plate	3 mL	0.1, 0.25, 1 mg/well	2, 5, 20 µg/10^5 cells	Flow cytometry: Neutrophil size and granularity; Neutrophil viability: Annexin-V/PI staining; CD cell surface markers; Cytokine production; Particle interference
**Poirier *et al.*,** [43]	Human neutrophils	1 x10^7 cells/mL	96 well plate detailed for Apoptosis assay only	No detailed	10, 100 µg/mL	0.1, 1 µg/10^5 cells	Neutrophil viability: morphological analysis; LDH release; Cytokine production; MMP-9 release
**Liz *et al.*,**[25]	Human neutrophils	1 x10^7 cells/mL	Not detailed	No detailed	5, 10, 25 µg/ mL,	0.05, 0.1, 0.25 µg /10^5 cells	Flow cytometry: Neutrophil size and granularity; Neutrophil viability: Annexin-V/CD16; TEM; ROS production; Caspase cleavage
**Johnston *et al.*,**[57]	Differentiated HL-60 cells (neutrophil-like)	1 x10^5 cells/well	96 well plate	100 µL	3.9, 7.9, 15.6, 125 µg/mL (cytotoxicity assay only)	0.39, 0.78, 1.56 µg/10^5 cells	Cytotoxicity Assay: alamar blue; Cell viability: Annexin-V; ROS production Cytokine production
**Saores *et al.*,** [34]	Human neutrophils	2 x10^6 cells/mL	Not detailed	No detailed	25, 50, 100 µg/mL	1.25, 2.5, 5 µg/10^5 cells	Cell viability: Trypan blue exclusion; PI staining; Neutrophil oxidative burst Neutrophil morphology; TEM

Supplementary figure 1:

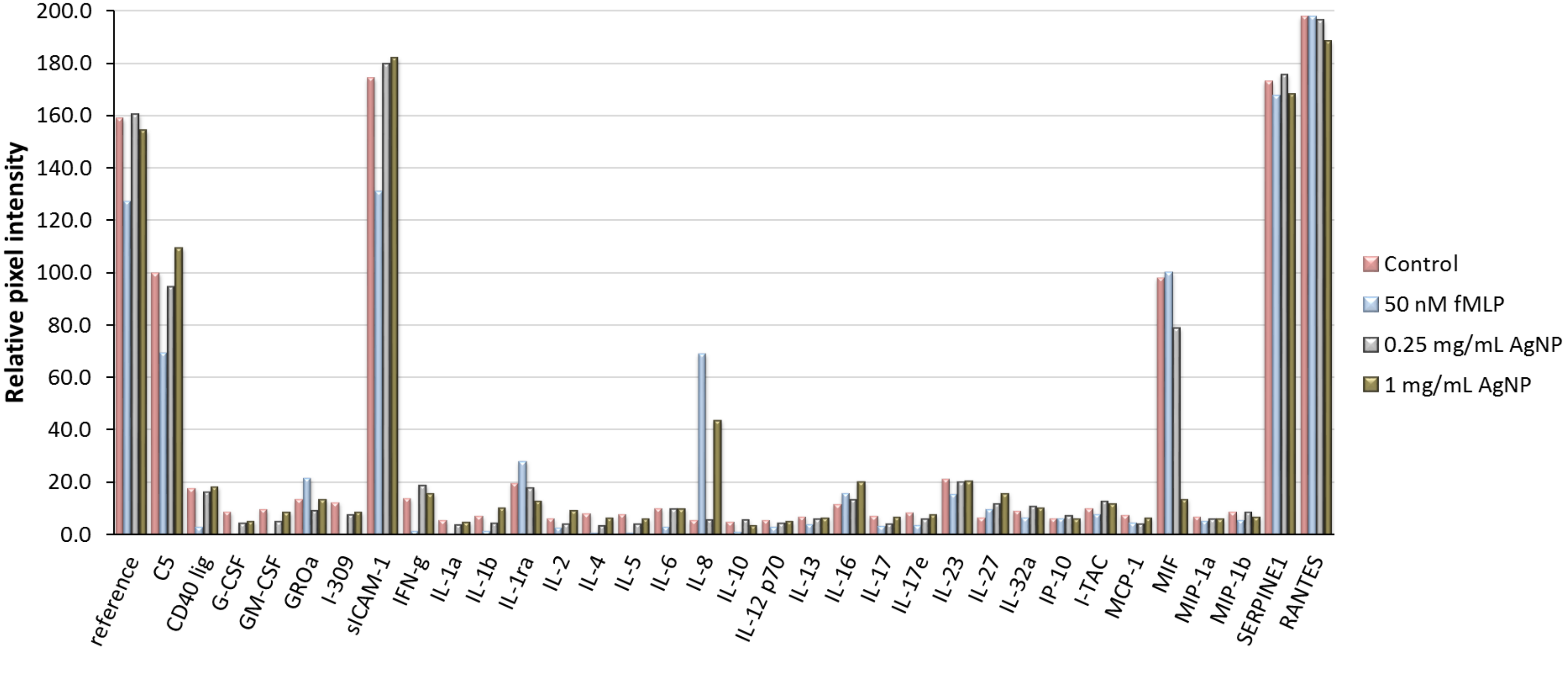

Supplementary figure 2:

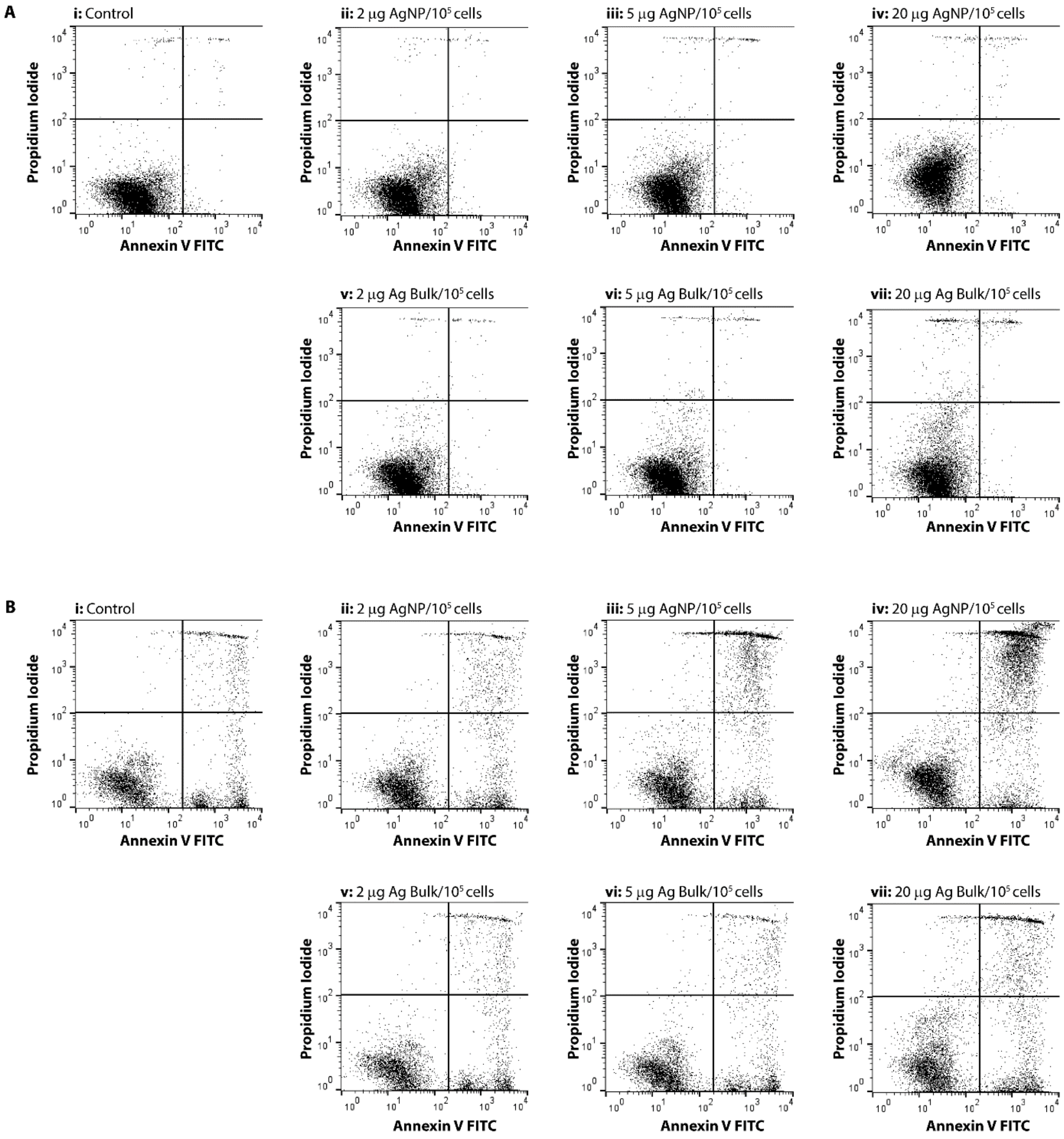

Figure legends:

Supplementary Table 1: Comparison of the final silver particle concentrations obtained by others using *in vitro* models of neutrophils. CD: cluster of differentiation; LDH: lactate dehydrogenase; MMP: matrix metalloproteinase; PI: propidium iodide; ROS: reactive oxygen species; TEM: transmission electron microscopy.

Supplementary Figure 1: Profiling the cytokines released from human neutrophils in response to silver nanoparticles. Neutrophils were treated with 0.25 or 1 mg/mL AgNP or 50nM fMLP for 4hr at 37°C before the culture medium was removed and the profile of cytokine release was assayed by profiler array (n=1). The pixel intensity of each spot was measured using ImageJ and the average of the duplicate cytokine spots was plotted. *Ref:* reference spot.

Supplementary Figure 2: Analysis of human neutrophil viability via flow cytometry. Representative images of AnV and PI stained untreated (Con; panel i), 2, 5 or 20 µg AgNP (NP; panels ii-iv) or 2, 5 or 20 µg Ag Bulk particle (panel v-iiv) treated human neutrophils (µg/per 10^5 cells). Neutrophils were incubated at 37°C for 4 hr (A) or 20hr (B) in the presence or absence of particles before viability was assessed via AnV and PI staining and flow cytometry, counting 10000 gated events. Quadrants applied to AnV/PI stained cells to discriminate between non-apoptotic, early apoptotic, late-apoptotic and necrotic cells (right hand panel) are shown. The number of events per quadrant was calculated as a percentage of total number of gated events and is expressed graphically in Figure 2.

References:

57. Johnston H, Brown D, Kanase N, Euston M, Gaiser B, Robb C, Dyrynda E, Rossi A, Brown E, Stone V. Mechanism of neutrophil activation and toxicity elicited by engineered nanomaterials. *Toxicology in Vitro* 29(5) 1172-1184 (2015).
